# Model Selection Emphasises the Importance of Non-Chromosomal Information in Genetic Studies

**DOI:** 10.1371/journal.pone.0117014

**Published:** 2015-01-27

**Authors:** Reda Rawi, Mohamed El Anbari, Halima Bensmail

**Affiliations:** 1 Computational Science and Engineering Center, Qatar Computing Research Institute, Doha, Qatar; 2 Division of Biomedical Informatics, Sidra Medical and Research Center, Doha, Qatar; Centers for Disease Control and Prevention, UNITED STATES

## Abstract

Ever since the case of the missing heritability was highlighted some years ago, scientists have been investigating various possible explanations for the issue. However, none of these explanations include non-chromosomal genetic information. Here we describe explicitly how chromosomal and non-chromosomal modifiers collectively influence the heritability of a trait, in this case, the growth rate of yeast. Our results show that the non-chromosomal contribution can be large, adding another dimension to the estimation of heritability. We also discovered, combining the strength of LASSO with model selection, that the interaction of chromosomal and non-chromosomal information is essential in describing phenotypes.

## Introduction

Genome-wide association studies (GWAS) have contributed to the identification of many human loci associated with a wide range of complex traits, such as height, intelligence or diseases such as obesity, type 2 diabetes and age-related macular degeneration. However, GWAS do not explain the whole story of the observed heritability of these traits [1, 2]. Explanations for this missing heritability include—amongst others—variants with effects too small to be identified with statistical significance [3], variant interactions that cannot be detected with current estimates [4], rare variants not identified by GWAS [3], and epigenetic effects [5–7].

Interestingly, non-chromosomal genetic information has not yet been taken into account when estimating heritability, although there is evidence for effects on the phenotype arising from cytoplasmic elements in many different organisms. For instance, Cadwell et al. [8] showed in their work on a mouse model that the interaction between a specific virus infection and a mutation in Crohn’s disease susceptibility gene *Atg16L1* induces intestinal pathologies.

Recently, our collaborators from MIT have designed an experiment using yeast where both sources of information, chromosomal and non-chromosomal were controlled in order to observe the phenotype of a single chromosomal polymorphism in the presence of different cytoplasmic elements [9]. They showed that the source of the mitochondrial genome, and the presence or absence of a dsRNA virus, both affect the phenotype of chromosomal variants [9].

Unfortunately, their statistical analysis had some limitations. Firstly, they split the data into training and test sub-samples (1/10 of the data held-out for testing) in order to conduct ten-fold cross validation. This appears to be the wrong approach, given that the sample sizes (see supplementary S1 Table) for the different gene deletion experiments range from 9 to 20. Secondly, they computed the coefficient of determination (*R* ^2^), here used as metric for recovered heritability, for three different models that consider (i) only the chromosomal mutation, (ii) the effects of the chromosomal mutation and non-chromosomal information, and (iii) both the effect of the chromosomal mutation and non-chromosomal information as well as their interaction. They inferred—exclusively from the gain in *R* ^2^—that non-chromosomal information and interaction effects substantially contribute to the heritability. However, it is well known that *R* ^2^ values may increase with increasing number of explanatory variables, and, hence, can not be exclusively applied to meaningfully compare models with different number of variables.

In this study, we applied more sophisticated statistical means and models, such as the adjusted coefficient of determination ($R_{a}^{2}$), a different cross validation strategy and a combination of Least Absolute Shrinkage and Selection Operator (LASSO) [10] with the Bayesian information criterion (BIC) [11, 12], as well as the recently introduced LASSO for hierarchical interactions [13], to detect the effects of non-chromosomal modifiers on the heritability of a trait. Our results confirm the importance of non-chromosomal information and its interaction with chromosomal mutations, when using both LASSO and BIC as well as LASSO for hierarchical interactions.

## Results

Previous work studied two inherited non-chromosomal modifiers. First, the presence and absence of the endogenous dsRNA yeast “killer” virus that is transmitted by mitosis and meiosis [14, 15], and second, the mitochondrial diversity. Strains were either constructed with ([kil-k]) or without ([kil-0]) the virus, that may spontaneously be lost and transition from [kil-k] to [kil-0]. Hence, its presence was constantly controlled by either a petri plate assay, or by detection of dsRNA on a gel. Furthermore, experiments were designed using either S288c ([rho+]^S288c^) or Sigma mitochondria ([rho+]^Sigma^). The mitochondria differ strongly in their genomes, with about 2–3 SNPs per kilobase and ten times more insertions and deletions compared to the chromosomal genome. The studied model chromosomal modifiers were gene deletions in the yeast strains S288c and Sigma [16]. Previous studies already detected several mutations with lethal or slow growth phenotypes in one strain, but not in the other [16]. A detailed description of the experiments can be found in Edwards et al. [9]. A summary of the colony size measurements is provided in S1 Table (see supplementary).

### Non-chromosomal information explains increased heritability

We analysed the effects of non-chromosomal modifiers on growth phenotypes of chromosomal variants using raw data from Edwards et al. [9], with *c*_*ij*_ as the colony size of controlled genotype i in replicate j. We applied a variance-stabilising Box-Cox transform on the *c*_*ij*_ values with exponent of 0.25 to obtain normalised values *y*_*ij*_, as this parameter choice gave the least-correlated means and variances in the analysed sets (according to Edwards et al. [9]). Next, we split the data (for each genotype i separately) into equally sized training and test sub-samples and applied the following linear models, (i) *Y* = *β*_0_ + *β*_1_ *X*_1_ + *ϵ* (*simple*), (ii) *Y* = *β*_0_ + *β*_1_ *X*_1_ + *β*_2_ *X*_2_ + *ϵ* (*additive*), and (iii) *Y* = *β*_0_ + *β*_1_ *X*_1_ + *β*_2_ *X*_2_ + *β*_3_ *X*_1_ *X*_2_ + *ϵ* (*interaction*), with *Y* = (*y*_1_, …, *y*_*n*_)^*t*^ as the response vector, *ɛ* = (*ɛ*_1_, …, *ɛ*_*n*_)^*t*^ ∼ *N*(0, *σ* ^2^ *I*_*n*_) as the noise vector, *X*_*j*_ as the *j*th predictor for *j* = 1, …, *p*, and *β* = (*β*_1_, …, *β*_*p*_)^*t*^ as the vector of parameters of interest to be estimated. Each *β*_*j*_, *j* = 1, …, *p* represents the association between the variable *X*_*j*_ and the response *Y*.

The *simple* model considered only the chromosomal mutation, whereas the *additive* and *interaction* model considered both chromosomal and non-chromosomal effects. The *interaction* model includes the interaction between chromosomal and non-chromosomal effects. We then calculated $R_{a}^{2}$ values (similar to Edwards et al. [9]), for the three different models. We applied $R_{a}^{2}$, a modification of *R* ^2^, because it is capable of handling the inflation of *R* ^2^, when comparing different models.

**Fig 1:**
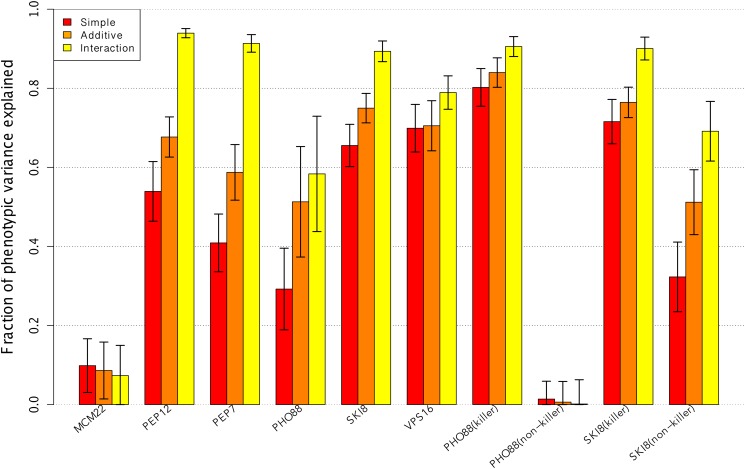
Non-chromosomal information enhances the fraction of phenotypic variance explained. Three linear models with different complexity are applied to measure the fraction of phenotypic variance. The first model (*simple*) includes only the gene deletion status (red), the second model (*additive*) considers the gene deletion status and non-chromosomal elements (orange), and finally the third model (*interaction*) includes both chromosomal and non-chromosomal elements as well as their interaction (yellow). The fraction of phenotypic variance is thereby approximated by the average coefficient of determination ($R_{a}^{2}$) of 1000 randomly sampled sub-sets. Aside from the control *MCM22* and the gene deletion *PHO88(non-killer)* experiment, model accuracy increases considerably when non-chromosomal information is included and much more when the interaction is taken into account.

In Fig. 1 we illustrated the fractions of phenotypic variances (y-axis) for ten single gene deletions (x-axis) with the three different models. In order to ensure the stability of the $R_{a}^{2}$ values we repeated the procedure of splitting the data into training and test sub-samples and calculating the $R_{a}^{2}$ 1000 times. We then plotted the average $R_{a}^{2}$ as bar heights. The error bars show the standard deviation across the 1000 sampled test sets. Bars representing the *simple* model are illustrated in red, *additive* in orange and the *interaction* model is shown in yellow. Aside from the control *MCM22* and the gene deletion *PHO88(non-killer)* experiment, it is clear that all experiments had a noticeably increase in model accuracy when including non-chromosomal and interaction effects.

Additional to the $R_{a}^{2}$, we computed, for each of the three linear models, the mean squared error (MSE) (see Material and Methods) to compare their performances. We repeated the procedure, as for the $R_{a}^{2}$, 1000 times and considered the model with the lowest MSE as the best for the given test sub-samples. In Table 1 we show, for each gene deletion experiment, the frequency of the chosen linear models according to their MSEs. Again we observed that, aside from the control *MCM22* and the gene deletion experiment *PHO88(non-killer)*, the *interaction* model is chosen in most cases, which emphasises the importance of the non-chromosomal information and its interaction with chromosomal mutations.

**Table 1:** Frequency of selected linear models according to their MSE within 1000 modelling repeats.

**Gene deletion**	***simple***	***additive***	***interaction***
*MCM22*	908	81	11
*PEP12*	0	0	1000
*PEP7*	0	0	1000
*PHO88*	60	121	819
*SKI8*	3	13	984
*VPS16*	32	34	934
*PHO88_MITO_KILL*	35	31	934
*PHO88_MITO_NO-KILL*	606	212	182
*SKI8_MITO_KILL*	18	4	978
*SKI8_MITO_NO-KILL*	1	18	981

Each row shows the frequency of selected (according to the MSE) linear models for a gene deletion experiment within 1000 modelling repeats. The interaction model is, aside from the control *MCM22* and the *PHO88(non-killer)* experiment, chosen in most cases as the best.

Despite these results indicating the importance of non-chromosomal information, we applied LASSO along BIC to verify our findings.

### LASSO and BIC

We analysed the effects of the three predictors (*X*_1_, *X*_2_ and *X*_1_ *X*_2_) using LASSO alongside BIC. We take advantage of the fact that model selection criteria extracts from a set of candidate models (in our case the *simple*, *additive* and *interaction* models) those that best describe a given dataset. One advantage of LASSO over simple linear models is that the regression and model selection can be applied in a single procedure.

As in the previous approach, we performed 1000 LASSO regressions with BIC model selection for each gene deletion experiment. We then studied the complexity of the BIC-selected models that best describe the given data. In Table 2 we summarise the different model sizes selected during 1000 modelling repeats. If we disregard the gene deletions *MCM22* and *PHO88(non-killer)*, most experiments require two or three predictors to explain the data. An exception is the *PHO88* gene deletion experiment, where, in 618 out of 1000 modelling repeats, one predictor is sufficient. The predictor representing the interaction between chromosomal and non-chromosomal modifiers was preferentially chosen by BIC.

**Table 2:** Complexity of BIC selected statistical models.

**Gene deletion**	**Intercept**	**One Predictor**	**Two Predictors**	**Three Predictors**
*MCM22*	429	544	27	0
*PEP12*	0	0	451	549
*PEP7*	0	11	442	547
*PHO88*	0	618	155	227
*SKI8*	0	0	992	8
*VPS16*	0	13	576	411
*PHO88(killer)*	0	2	983	15
*PHO88(non-killer)*	883	106	10	1
*SKI8(killer)*	0	0	891	109
*SKI8(non-killer)*	0	0	961	39

Model selection using BIC was applied in each of the 1000 modelling repeats for all gene deletion experiments. Except for the control *MCM22* and the *PHO88(non-killer)* experiment most cases require two or three predictors in order to describe the given data best. However, in the *PHO88* deletion case only one predictor is necessary in 618 of 1000 modelling cases.

Furthermore, we analysed the frequency of predictors representing chromosomal (*X*_1_) and non-chromosomal effects (*X*_2_) as well as their interaction (*X*_1_ *X*_2_) within the 1000 procedures (see Table 3). Excluding the cases *MCM22* and *PHO88(non-killer)*, the predictors *X*_1_ and *X*_1_ *X*_2_ are selected in most cases. Regarding the gene deletion *PHO88* experiment, the *interaction* term (*X*_1_ *X*_2_) is chosen more often as the other predictors.

**Table 3:** Frequency of BIC selected predictors representing chromosomal (*X*_1_) and non-chromosomal effects (*X*_2_) as well as their interaction (*X*_1_ *X*_2_) within 1000 modelling repeats.

**Gene deletion**	***X*_1_**	***X*_2_**	***X*_1_*X*_2_**
*MCM22*	518	13	67
*PEP12*	991	558	1000
*PEP7*	907	629	1000
*PHO88*	379	237	993
*SKI8*	1000	8	1000
*VPS16*	997	414	987
*PHO88(killer)*	1000	5	998
*PHO88(non-killer)*	89	26	14
*SKI8(killer)*	1000	109	1000
*SKI8(non-killer)*	1000	39	1000

Aside from the *MCM22* and *PHO88(non-killer)* gene deletion experiments, the predictors representing the chromosomal (*X*_1_) as well as the interaction effect (*X*_1_ *X*_2_) are selected in almost all modelling processes. An exception is the *PHO88* deletion experiment where only the interaction predictor (*X*_1_ *X*_2_) is most frequently selected.

As for the previous method, these results highlighted the importance of the interaction between chromosomal and non-chromosomal modifiers. However, we discovered that LASSO selected the main or interaction effects arbitrarily (not only for gene deletion experiment *PHO88*), while it is a general good statistical practice to include interaction effects only if the main effects are also in the model. Therefore, we applied the recently introduced LASSO for hierarchical interaction [13].

### LASSO for hierarchical interactions

In order to use LASSO for hierarchical interactions, we used the R software package *hierNet* [17, 18] that fits interaction models with the restriction that the interaction between two variables is only included if both variables are included as main effects (strong hierarchy). The analysis procedure consisted of the following steps. First, we fitted several LASSO models with different values of the regularisation parameter *λ* (function *hierNet.path*) to the data. Second, we applied a cross validation (function *hierNet.cv*) and chose the model with the largest value of *λ* such that the error is within 1 standard error of the minimum. We repeated this procedure 1000 times and analysed the complexity of the resulting models as before.

Interestingly, we identified that in most cases, aside from the experiments *MCM22* and *PHO88(non-killer)*, three predictors (*X*_1_, *X*_2_ and *X*_1_ *X*_2_) are required to best describe the data (see S2 Table). An exception is the gene deletion experiment *PHO88*, where only around half of the 1000 modelling repeats required three predictors. This recently developed LASSO approach confirmed our earlier findings that non-chromosomal information and its interaction with chromosomal mutation is important.

## Discussion

Our analyses revealed that the phenotype of a chromosomal mutation may be affected by non-chromosomal elements such as mitochondria and viral state. We also showed that the introduction of non-chromosomal information and its interaction with chromosomal elements considerably enhanced the fraction of explained phenotypic variance of a trait, which is ensured by conserving the chromosomal contribution and the environment, whilst changing the non-chromosomal effects. Previous studies [19–23], that crossed strains carrying a dsRNA virus with virus-free strains as S288c [24], may have also been affected by non-chromosomal elements or their interaction with chromosomal ones, although we cannot prove it without repeating the experiments and analyses.

However, it is well known that the coefficient of determination, here used as metric for recovered heritability, is prone to an increase when adding more variables to the statistical model. Hence, we could not exclude that the gain in $R_{a}^{2}$ arose only from gain in explained heritability.

Due to this fact, we applied model selection criteria BIC to investigate the importance of both chromosomal and non-chromosomal information, as well as their interaction in describing colony size data. BIC highlighted that not only chromosomal mutations, but also the interactions between chromosomal and non-chromosomal elements, such as mitochondria and dsRNA virus are important. The mitochondrial background plays a crucial role in the *PHO88* gene deletion case, where cells grow faster with a Sigma mitochondria background ([kil-k] [rho+]^Sigma^) compared to cells with a S288c mitochondria ([kil-k] [rho+]^S288c^). With BIC model selection, the *interaction* term (*X*_1_ *X*_2_) is sufficient to describe the data for this gene deletion experiment in two out of three cases.

Furthermore, we examined LASSO with hierarchical interactions. This approach, unlike ordinary LASSO, prevents the inclusion of interaction effects unless the main effects are also included. We identified for most cases, that not only non-chromosomal, but also its interaction with chromosomal effects, is essential to best describe the colony size data. For the *PHO88* gene deletion case, we identified that the interaction effect is chosen as important in about half the modelling repeats.

In summary, all applied statistical methods point to the fact that non-chromosomal modifiers, and the interaction effects of chromosomal and non-chromosomal elements, account for a substantial fraction of phenotypic variance of growth rates in yeast.

## Materials and Methods

### Data

The raw data measurements and the analysis script (R code) can be found in the supplementary section.

### Regularisation models

We consider the standard multiple linear regression model with *n* observations and *p* explanatory variables (predictors)

$$Y=\beta_{0}+\beta_{1}X_{1}+\beta_{2}X_{2}+\ldots +\beta_{p}X_{p}+\epsilon ,$$ (1)

where *Y* = (*y*_1_, …, *y*_*n*_)^*t*^ is the response vector, *ɛ* = (*ɛ*_1_, …, *ɛ*_*n*_)^*t*^ ∼ *N*(0, *σ* ^2^ *I*_*n*_) is the noise vector; for *j* = 1, …, *p*, *X*_*j*_ represents the *j*th predictor and *β* = (*β*_1_, …, *β*_*p*_)^*t*^ is the vector of parameters of interest to be estimated; each *β*_*j*_, *j* = 1, …, *p* represents the association between the variable *X*_*j*_ and the response *Y*. Given estimates ${\hat{\beta }}_{1},\ldots ,{\hat{\beta }}_{p}$, we can make predictions using the formula

$$\hat{Y}={\hat{\beta }}_{0}+{\hat{\beta }}_{1}X_{1}+{\hat{\beta }}_{2}X_{2}+\ldots +{\hat{\beta }}_{p}X_{p}.$$ (2)

### Coefficient of determination (*R* ^2^)

Define $\text{TSS}=\sum_{i=1}^{n}{\left(y_{i}-\bar{y}\right)}^{2}$ as the *total sum of squares* and the *residual sum of squares* (RSS) as $\text{RSS}=\sum_{i=1}^{n}{\left(y_{i}-{\hat{y}}_{i}\right)}^{2}$. The coefficient of determination *R* ^2^ or the percentage of variance explained is defined as

$$R^{2}=1-\frac{\sum_{i=1}^{n}{\left(y_{i}-{\hat{y}}_{i}\right)}^{2}}{\sum_{i=1}^{n}{\left(y_{i}-\bar{y}\right)}^{2}}=1-\frac{\text{RSS}}{\text{TSS}}.$$ (3)

### Adjusted coefficient of determination ($R_{a}^{2}$)

Since RSS always decreases as more variables are added to the model, *R* ^2^ always increases as more variables are added. For a least squares model with *q* variables, the $R_{a}^{2}$ statistic is calculated as

$$R_{a}^{2}=1-\frac{\text{RSS}/(n-q-1)}{\text{TSS}/(n-1)}.$$ (4)

Maximising $R_{a}^{2}$ is equivalent to minimising RSS/(*n* − *q* − 1). While RSS always decreases as the number of variables increases, RSS/(*n* − *q* − 1) may increase or decrease, due to the presence of *q* in the denominator. Hence, the $R_{a}^{2}$ statistic can be used for selecting among a set of models that contains different number of variables.

### MSE

If we have enough observations, we can divide our data set into two parts: a training set of size *n*_*train*_, on which the model is fitted, and a test set of size *n*_*test*_ for evaluation of the performance. A measure of prediction performance commonly used is the *mean squared error* (MSE) on the test set, and it is defined as

$$\text{MSE}=\frac{1}{n_{test}}\sum_{i=1}^{n_{test}}{\left(y_{i,test}-{\hat{y}}_{i,test}\right)}^{2},$$ (5)

where *y*_*i*, *test*_ and ${\hat{y}}_{i,test}$ are respectively the real and predicted values of the response *Y* in the test data.

### LASSO

The LASSO coefficients, ${\hat{\beta }}_{\lambda }^{L}$, minimises the quantity

$$\text{RSS}+\lambda \sum_{j=1}^{p}|\beta_{j}|.$$ (6)

The LASSO technique penalises the regression coefficients using an *l*_1_ norm. It shrinks the coefficients towards zero. In addition, the *l*_1_ penalty has the effect of forcing some of the coefficient to be exactly equal to zero when the tuning parameter *λ* is sufficiently large. Hence, the LASSO estimates the coefficients and performs variable selection in a single procedure. The choice of the tuning parameter *λ* is critical and can be performed using cross validation.

### BIC

For the least squares model with *q* predictors, the BIC is, up to irrelevant constants, given by

$$\text{BIC}=\frac{1}{n}\left(\text{RSS}+\log\left(n\right)q{\hat{\sigma }}^{2}\right),$$ (7)

where ${\hat{\sigma }}^{2}$ is an estimate of the variance of *ɛ*. We select the model that has the lowest BIC value.

### LASSO for hierarchical interactions

Bien el al. [13] proposed an interesting approach. They consider the *two-way* interaction model

$$Y=\beta_{0}+\sum_{j=1}^{p}\beta_{j}X_{j}+\frac{1}{2}\sum_{j\neq k}\theta_{jk}X_{j}X_{k}+\epsilon .$$ (8)

The additive part is called the *main effect*, while the quadratic part is called the *interaction* terms. The goal is to estimate *β* ∈ ℝ^*p*^ and Θ ∈ ℝ^*p* × *p*^, where Θ = Θ^*t*^ and *θ*_*jj*_ = 0 for *j* = 1, …, *p*. This is done using an *all-pairs Lasso* criterion, which has the following form

$${\text{Minimize}}_{\beta_{0}\in R,\beta \in R^{p},\Theta \in R^{p\times p}}\frac{1}{2}\sum_{i=1}^{n}{\left(y_{i}-\beta_{0}-x_{i}^{t}\beta -\frac{1}{2}x_{i}^{t}\Theta x_{i}\right)}^{2}+{\lambda ∥\beta ∥}_{1}+\frac{\lambda }{2}{∥\Theta ∥}_{1},$$ (9)

where $\|\beta {\|}_{1}=\sum_{j=1}^{p}|\beta_{j}|$, ‖Θ‖_1_ = ∑_*j* ≠ *k*_∣*θ*_*jk*_∣, *x*_*i*_ is the observed value of *X*_*i*_, *β*_0_ is the intercept, and *λ* is a positive tuning parameter that can be estimated using cross validation. The method produces sparse interaction models that honour the hierarchy restriction that an interaction is only included in a model if one or both variables are marginally important.

## Supporting Information

S1 Table Summary of colony measurements. (PDF)

S2 Table Complexity of best statistical models chosen by LASSO for hierarchical interactions. (PDF)

S3 Table Frequency of predictors (X1, X2 and X1X2.) within 1000 modelling repeats using LASSO for hierarchical interactions. (PDF)

S1 Script R analysis script. (R)

S1 Data Raw data measurements. (CSV)

## References

[1] Eichler EE, Flint J, Gibson G, Kong A, Leal SM, et al (2010) Missing heritability and strategies for finding the underlying causes of complex disease. Nature reviews Genetics 11: 446–450. 10.1038/nrg2809 20479774 PMC2942068

[2] Manolio Ta, Collins FS, Cox NJ, Goldstein DB, Hindorff La, et al (2009) Finding the missing heritability of complex diseases. Nature 461: 747–753. 10.1038/nature08494 19812666 PMC2831613

[3] Bloom JS, Ehrenreich IM, Loo WT, Lite TLVo, Kruglyak L (2013) Finding the sources of missing heritability in a yeast cross. Nature 494: 234–237. 10.1038/nature11867 23376951 PMC4001867

[4] Zuk O, Hechter E, Sunyaev SR, Lander ES (2012) The mystery of missing heritability: Genetic interactions create phantom heritability. Proceedings of the National Academy of Sciences of the United States of America 109: 1193–1198. 10.1073/pnas.1119675109 22223662 PMC3268279

[5] Slatkin M (2009) Epigenetic inheritance and the missing heritability problem. Genetics 182: 845–850. 10.1534/genetics.109.102798 19416939 PMC2710163

[6] Rassoulzadegan M, Grandjean V, Gounon P, Vincent S, Gillot I, et al (2006) RNA-mediated non-mendelian inheritance of an epigenetic change in the mouse. Nature 441: 469–474. 10.1038/nature04674 16724059

[7] Nadeau JH (2009) Transgenerational genetic effects on phenotypic variation and disease risk. Human molecular genetics 18: R202–10. 10.1093/hmg/ddp366 19808797 PMC2758712

[8] Cadwell K, Patel KK, Maloney NS, Liu TC, Ng ACY, et al (2010) Virus-plus-susceptibility gene interaction determines Crohn’s disease gene Atg16L1 phenotypes in intestine. Cell 141: 1135–1145. 10.1016/j.cell.2010.05.009 20602997 PMC2908380

[9] Edwards MD, Symbor-Nagrabska A, Dollard L, Gifford DK, Fink GR (2014) Interactions between chromosomal and nonchromosomal elements reveal missing heritability. Proceedings of the National Academy of Sciences of the United States of America 111: 7719–7722. 10.1073/pnas.1407126111 24825890 PMC4040555

[10] Tibshirani R (1996) Regression Shrinkage and Selection via the Lasso. Journal of the Royal Statistical Society (Series B) 58: 267–288.

[11] Schwarz G (1978) Estimating the Dimension of a Model. The Annals of Statistics 6: 461–464. 10.1214/aos/1176344136

[12] Bogdan M, Ghosh JK, Doerge RW (2004) Modifying the Schwarz Bayesian information criterion to locate multiple interacting quantitative trait loci. Genetics 167: 989–999. 10.1534/genetics.103.021683 15238547 PMC1470914

[13] Bien J, Taylor J, Tibshirani R (2013) A lasso for hierarchical interactions. The Annals of Statistics 41: 1111–1141. 10.1214/13-AOS1096 26257447 PMC4527358

[14] Magliani W, Conti S, Gerloni M, Bertolotti D, Polonelli L (1997) Yeast killer systems. Clinical microbiology reviews 10: 369–400. 9227858 10.1128/cmr.10.3.369 PMC172926

[15] Schmitt MJ, Breinig F (2006) Yeast viral killer toxins: lethality and self-protection. Nature reviews Microbiology 4: 212–221. 10.1038/nrmicro1347 16489348

[16] Dowell RD, Ryan O, Jansen A, Cheung D, Agarwala S, et al (2010) Genotype to phenotype: a complex problem. Science (New York, NY) 328: 469 10.1126/science.1189015 PMC4412269 20413493

[17] R Core Team (2014) R: A Language and Environment for Statistical Computing. R Foundation for Statistical Computing, Vienna, Austria URL http://www.R-project.org/.

[18] Bien J, Tibshirani R (2014) hierNet: A Lasso for Hierarchical Interactions. URL http://CRAN.R-project.org/package=hierNet. R package version 1.6. 10.1214/13-AOS1096 PMC4527358 26257447

[19] Ben-Ari G, Zenvirth D, Sherman A, David L, Klutstein M, et al (2006) Four linked genes participate in controlling sporulation efficiency in budding yeast. PLoS genetics 2: e195 10.1371/journal.pgen.0020195 17112318 PMC1636695

[20] Sinha H, David L, Pascon RC, Clauder-Münster S, Krishnakumar S, et al (2008) Sequential elimination of major-effect contributors identifies additional quantitative trait loci conditioning high-temperature growth in yeast. Genetics 180: 1661–1670. 10.1534/genetics.108.092932 18780730 PMC2581965

[21] Steinmetz LM, Sinha H, Richards DR, Spiegelman JI, Oefner PJ, et al (2002) Dissecting the architecture of a quantitative trait locus in yeast. Nature 416: 326–330. 10.1038/416326a 11907579

[22] Deutschbauer AM, Davis RW (2005) Quantitative trait loci mapped to single-nucleotide resolution in yeast. Nature genetics 37: 1333–1340. 10.1038/ng1674 16273108

[23] Kim HS, Fay JC (2009) A combined-cross analysis reveals genes with drug-specific and background-dependent effects on drug sensitivity in Saccharomyces cerevisiae. Genetics 183: 1141–1151. 10.1534/genetics.109.108068 19720856 PMC2778966

[24] Fink GR, Styles CA (1972) Curing of a killer factor in Saccharomyces cerevisiae. Proceedings of the National Academy of Sciences of the United States of America 69: 2846–2849. 10.1073/pnas.69.10.2846 4562744 PMC389659

