# Supplementary material for: Model Selection Emphasises the Importance of Non-Chromosomal Information in Genetic Studies
Source: PLoS One. 2015 Jan 27;10(1):e0117014. doi: 10.1371/journal.pone.0117014 (PMC4308103; doi:10.1371/journal.pone.0117014)
Supplement: S1 Table — (PDF) [file pone.0117014.s001.pdf]

Supplementary information for:

Model selection emphasises the importance of non-chromosomal information in genetic studies

Reda Rawi[1], Mohamed El Anbari[1,2], Halima Bensmail[1,*]

1 Computational Science and Engineering Center, Qatar Computing Research Institute, Doha, Qatar

2 Division of Biomedical Informatics, Sidra Medical and Research Center, Doha, Qatar

∗ E-mail: hbensmail@qf.org.qa

Table S1: Summary of colony measurements.

Gene	Non-chromosomal element	Experiment type	Knockout?	Killer	S288c mito.?	Measured colonies	Average size, pixels	Colony size SD, pixels
PEP12	Killer virus	Deletion vs.	1	1	0	18	104.1	69.3
		killer	1	0	0	15	2,638.9	885.8
			0	1	0	18	5,149.8	1,090.1
			0	0	0	18	4,105.9	894.3
PEP7	Killer virus	Deletion vs.	1	1	0	14	145.1	154.5
		killer	1	0	0	18	3,563.7	1,028.4
			0	1	0	13	6,744.8	1,552.7
			0	0	0	18	5,185.1	1,909.1
PHO88	Killer virus	Deletion vs.	1	1	0	14	183,4	73.5
		killer	1	0	0	14	361.9	40.2
			0	1	0	13	395.2	74.1
			0	0	0	14	465.5	74.9
SKI7	Killer virus	Deletion vs.	1	1	0	15	0.0	0.0
		killer	1	0	0	16	4,394.4	807.8
			0	1	0	15	5,458.6	1,122.2
			0	0	0	15	4,896.6	834.1
SKI8	Killer virus	Deletion vs.	1	1	0	11	638.0	438.2
		killer	1	0	0	18	2,481.7	594.7
			0	1	0	12	5,973.8	644.3
			0	0	0	18	5,603.5	966.8
VPS16	Killer virus	Deletion vs.	1	1	1	18	1,355.4	380.4
		killer	1	0	0	14	2,806.1	1,139.7
			0	1	1	18	7,326.1	2,168.0
			0	0	0	14	5,869.4	2,329.6
PHO88	Mitochondrial	Deletion vs.	1	1	1	9	51.6	36.0
		mito. (killer)	1	1	0	12	184.3	84.1
			0	1	1	10	754.4	95.5
			0	1	0	12	704.3	144.1
SKI8	Mitochondrial	Deletion vs.	1	1	1	13	1,328.9	350.4
		mito. (killer)	1	1	0	10	288.8	225.7
			0	1	1	14	3,762.0	610.1
			0	1	0	13	4,434.6	303.1
SKI8	Mitochondrial	Deletion vs.	1	0	1	18	290.3	71.5
		mito. (non-killer)	1	0	0	16	117.1	44.0
			0	0	1	18	346.3	75.5
			0	0	0	16	344.1	85.9
MCM22	Killer virus	Deletion vs.	1	1	0	18	2,166.3	260.3
		killer	1	0	0	18	2,178.4	332.0
			0	1	0	18	2,356.2	246.7
			0	0	0	18	2,372.6	263.3
PHO88	Mitochondrial	Deletion vs.	1	0	1	18	4,389.7	721.3
		mito. (non-killer)	1	0	0	20	4,387.7	806.3
			0	0	1	16	4,462.4	661.6
			0	0	0	20	4,712.4	574.6
