# Supplementary material for: Model Selection Emphasises the Importance of Non-Chromosomal Information in Genetic Studies
Source: PLoS One. 2015 Jan 27;10(1):e0117014. doi: 10.1371/journal.pone.0117014 (PMC4308103; doi:10.1371/journal.pone.0117014)
Supplement: S2 Table — (PDF) [file pone.0117014.s002.pdf]

Supplementary information for:

Model selection emphasises the importance of non-chromosomal information in genetic studies

Reda Rawi[1], Mohamed El Anbari[1,2], Halima Bensmail[1,*]

1 Computational Science and Engineering Center, Qatar Computing Research Institute, Doha, Qatar

2 Division of Biomedical Informatics, Sidra Medical and Research Center, Doha, Qatar

∗ E-mail: hbensmail@qf.org.qa

Table S2: Complexity of best statistical models chosen by LASSO for hierarchical interactions.

Gene deletion	Intercept	1 Predictor	2 Predictors	3 Predictors
MCM22	943	55	1	1
PEP12	0	0	0	1000
PEP7	0	0	0	1000
PHO88	357	20	156	467
SKI8	0	3	0	997
VPS16	0	126	4	870
PHO88(killer)	0	44	3	953
PHO88(non-killer)	992	6	1	1
SKI8(killer)	0	12	0	988
SKI8(non-killer)	2	3	0	995

LASSO for hierarchical interactions was applied in each of the 1000 modelling repeats for all gene deletion experiments. Except for the control *MCM22* and the *PHO88(non-killer)* experiment most cases required three predictors in order to describe the given data best.
