# Supplementary material for: Model Selection Emphasises the Importance of Non-Chromosomal Information in Genetic Studies
Source: PLoS One. 2015 Jan 27;10(1):e0117014. doi: 10.1371/journal.pone.0117014 (PMC4308103; doi:10.1371/journal.pone.0117014)
Supplement: S3 Table — (PDF) [file pone.0117014.s003.pdf]

Supplementary information for:

Model selection emphasises the importance of non-chromosomal information in genetic studies

Reda Rawi[1], Mohamed El Anbari[1,2], Halima Bensmail[1,*]

1 Computational Science and Engineering Center, Qatar Computing Research Institute, Doha, Qatar

2 Division of Biomedical Informatics, Sidra Medical and Research Center, Doha, Qatar

∗ E-mail: hbensmail@qf.org.qa

Table S3: Frequency of predictors (X_1,X_2 and X_1X_2.) within 1000 modelling repeats using LASSO for hierarchical interactions.

Gene deletion	X_1	X_2	X_1X_2
MCM22	57	2	1
PEP12	1000	1000	1000
PEP7	1000	1000	1000
PHO88	640	626	467
SKI8	1000	997	997
VPS16	1000	874	870
PHO88(killer)	1000	956	953
PHO88(non-killer)	7	3	1
SKI8(killer)	1000	988	988
SKI8(non-killer)	998	995	995

Aside from the *MCM22* and *PHO88(non-killer)* gene deletion experiments, all variables (X_1,X_2 and X_1X_2) are selected in most modelling repeats. An exception is the *PHO88* deletion experiment where the interaction predictor (X_1X_2) is selected in only half of the 1000 repeats.
